# Practical Thoughts on Tooth-Drawing

**Published:** 1855-01

**Authors:** C. T. Cushman

**Affiliations:** Columbus, Ga.


### PRACTICAL THOUGHTS ON TOOTH-DRAWING.

### NO. IV.

BY C. T. CUSHMAN, D. D. S., COLUMBUS, GA.

"Il arrive tous les jours que l'on rencontre en ôtant une dent, de nouvelles difficultez que l'on ne peut pas prévoir."—FAUCHARD.

"All rules for extracting teeth must be subject to limitations, as circumstances will occasionally interfere, to throw the operator upon his own resources."—SNELL.

"As this operation, except in the eyes of those who delude themselves, is one of manual dexterity, it must not be believed that pathological and anatomical knowledge can supply the want of a practical hand."—DESIRABODE.

*A Case showing that a tooth may break in extraction, even when general appearances promise easy success.—Anomalous specimen.*

CASE. XIII. A negro girl, *aet.* 9, maxillary arches wide, teeth large and soft. The 1st left inferior molar with deep carious opening on posterior grinding surface, penetrating the pulp-chamber. Patient low seated, I applied the forceps, carefully forced it on the neck of the tooth, and made lateral and perpendicular traction.

The crown and anterior root only came away, a fracture at the *fourchure* leaving the remainder firm in its socket.

Tried a slender root-forceps—the hold chipped off, without effecting removal.

Applied the punch (lever) outside—loosened it some, but it was too firm for ejectment. The thumb-fulcrum, (pied-de-biche—"hinds foot") applied inside, brought it out. This posterior branch was equally divided into *two* large, diverging roots, which stood across the alveoli.

Here was a lower molar, with *three* roots firmly articulated, and the whole tooth of a soft degree of organization. Two of the roots accordingly broke off, sooner than they could withstand the necessary force of extraction. Reason sufficient, and valid. And such result will invariably happen to teeth that present less, or no very marked aberration of form, though extracted by persons of skill and experience, with approved and safe instruments. Although blame may be imputed in these instances, it does not properly attach itself.

*A case showing that neither the key nor forceps will always succeed.*

CASE XIV. Mr. H. *aet.* 30, nervous temperament.—April 21, 1852—been suffering a long time from periodontitis of the left inferior wisdom tooth. He applied to a physician, who broke it off so low with the key that he gave it up. He then came to me, and I tried forceps. By forcing it down, I still obtained what seemed to be a fair hold, but in trying to extract it, it broke off *again* on the line of grasp, without loosening.

The contiguous second molar was standing firm. I took the elevator, (*langue de carpe*) thrust the point obliquely down between it and the diseased roots and by a forcible turn, at once raised them out. The operation was painful, made so mostly by the highly inflamed condition of the gums and periosteum; but it was not more so than the two previous trials.

It is not every tooth that can withstand the hard pressure of forceps, or force of the key; particularly if it be necrosed;—but such tooth may not break under the elevator.

In using the elevator, or elevator-forceps against the upper wisdom teeth, there is danger of forcing off the coronal extremity of the alveolar ridge.

*The key for extracting the cuspid teeth.*

Case XV. (April 26, 1852.) A very old lady who was almost toothless, suffered acute periodontitis occasionally, from the root of the left inferior cuspid tooth. The crown was decayed away to the gum, the upper surface of the root, funnel shape, the sides so thin as to be almost certain to crush under the forceps.

The root was too firm set to be raised with the punch—the elevator would wound the gum, which was inflamed and puffed up around it. Her age was too great to admit of much patience on her part, or delay on mine, in the operation. Brevity was essential in this case.

Applied the key, hook inside—extracted the root instantly, and with but little pain, much to her delight.

There is no fact of the human character more remarkable than that the well-settled, honest convictions of the mind are sometimes supplanted by those of the very opposite nature.

I have known the time when (from reading and considering, more than from practical experience) I would have denounced the very title of this case as proclaiming a professional heresy. Under peculiar circumstances, I do not now so regard it at all.

*Ibid.*—No. 2, Case XVI. A middle aged man of nervous temperament, teeth naturally soft, and in wretched condition—lost nearly all, and wears several failing pivot teeth on which the lower incisors strike with aggravating and unresisted force.

Suffered three days severe pain in several or all. The left superior cuspid (with others) was to be extracted. Crown gone, gum receded some. As the root was slightly moveable, I was rash enough to oppose my own well-founded maxim, and promise that it would probably come out rather easy.

But the forceps broke it off, with a distressing *jar*, leaving it but little looser, and in no better condition for further effort.

I then applied the key, hook outside, and that quickly and nicely removed the root.

It has frequently come to my observation, that when the periosteal membrane of a tooth has for several years suffered inflammation, such teeth although seemingly loose to the touch, resists extractive force more stoutly than ordinarily. And when extracted, the investing tissue presents a peculiar appearance, somewhat that of being minutely broken up; instead of the usual smooth, laminal separation. May not this be the initial stage of Exostosis, or, bony consolidation. I have some pathological demonstrations of the latter, which are quite unmistakeable.

The key is the surest instrument for those young, and other *scary* persons who, perhaps never having had a tooth extracted, will detain you an hour before *making up their minds* to submit themselves and then allow you only one *grab.*

I remember a case in point, a young girl who baffled me nearly an hour and a half before allowing me to place the instrument upon an aching molar, the first right inferior. The crown was much hollowed by decay, but it probably might have been extracted with the forceps. I finally *nabbed* it with the forceps—there was a yell and a spring to get away. I felt the crown crushing, the hold giving way, and struggled to force the instrument further down, quite against her will—indeed it was much of a *tussle* between us.

I regretted it, and seldom if ever before attempted to operate under such disadvantage. Regretted it because I effected nothing, except to inflict some pain.

Now, with the key, sucess would have been more certain, because its rapidity of execution affords less chance for resistance. For *such* patients do I hold the instrument in particular reserve.

Some persons will suffer toothache many days, and as a desperate resort—to escape the torture of *another* night, will defer their long-contemplated visit to the dentist until the darkness of night is closing around. Under such unfavorable circumstances, with patients over-apprehensive, and restless, it is prudent to resort to palliatives, and to decline operating in the twilight, or by candle-light, unless it be a simple case.

The reputation of operators has unjustly suffered by the untoward results of attempting to extract teeth in such cases.

There are some persons whose teeth are so firm set and bones so solid, that the operation of extraction is a very serious and doubtful undertaking. The force necessary to dislodge such teeth is more than double that of the average amount; and unless it be exerted nearly on a line with its insertion, will be apt to break either the tooth or socket, and thus increase the patients calamity. Such teeth pertain to strong, athletic men, and those designated "mountain sprouts," who enjoy a high degree of bodily health and vigor. They are unmistakeable—large, thick, yellowish, and immoveable to the fingers.

Their roots which are long, broad, and curved, after the crowns are broken off by decay or otherwise, generally remain for many years without giving rise to pain or disease, and are also immoveable—apparently as solid as deep driven oak posts.

With acute toothache their owners suffer intensely, but are less subject to chronic attacks. The young dentist had best exhaust his palliatives on these patients, rather than confidently promise them easy and expeditious extraction.

I cannot respect those dentists who will not exemplify towards all patients, particularly those who are called to undergo this operation, the motto of "bear and forbear." The fee for tooth-drawing is, or ought to be, sufficient to ensure a liberal allowance of time, affability, and sympathy.

In extenuation of the indecisive, and sometimes provoking manner of timid patients, there should always be kept in view the traditionary *legends* of the operation, which, from the days of *Sampson* have invested it with such a terrible shroud of slaughter.

Enough of these horrors are founded on truth, but by far the greater bulk of them in fiction. They are injudiciously exaggerated, and kept alive by repetition.

It is thus that parents unwittingly intimidate their children; thus unnerving and unfitting them oftentimes, for the very endurance to which, as they must be aware, necessity will inevitably force them, in the rapid flight of time.

APPENDIX. The Editor of the *N. Y. Dental Recorder* (vol. VI. p. 181—1852) has manifestly aimed at my previous *Practical Thoughts*, the following comments:

"It is inexcusable carelessness when a dentist fractures a tooth through the sound part in attempting to extract it, unless it be the first or second time in his practice. It may be necessary to break a few teeth in the commencement of our operations, in order to learn their strength; but after obtaining that knowledge there is no excuse for ever breaking a tooth, except when it is so much decayed that the remaining strength cannot be correctly estimated. No more power should ever be expended upon a tooth, than it is capable of sustaining without fracture, and to what purpose have we practised for years if our experience has not taught us to avoid such gross carelessness? In these difficult cases if sufficient time is taken and the tooth is wrenched and turned in every direction long enough it will fianlly loosen in the socket so that it can be removed without fracture.

If the patient will not stand this, wherein is he benefitted by having his tooth fractured at the neck and the fangs left remaining in the socket?"

* * * "If he (the dentist) fractures the tooth, he merits and receives only his (patient's) anathemas."

In reply to which, I submit to the judgment of the profession the foregoing authoritative opinions, facts, and practical cases, as instances of professional experience.

I also give the opposing testimony of one of the *Recorder's* neighbors, Dr. Bridges of Brooklyn, well known to the profession:

"It is not possible to succeed in removing teeth in every case, without breaking them.

* * We sometimes encounter teeth that we cannot by fair means remove."—*Dental Mirror for* 1844, *p*. 8.

And I enter my protest against the *Recorder's* "anathemas" on those who disclaim that degree of skill which comes not short of infallibility. The positive languge of the commentator ( if such he be) certainly seems to bespeak it for himself. But, were he tested only in the cases instanced in this *series* and I here repeat, they are not of every day character. I am confident that his claim would have to be forfeited.

Will he please inform humble enquirers after truth, how every tooth can be " wrenched and turned in every direction long enough ?" ( We have read the similar directions given by ALBUCASIS in the eleventh century.) Wrenched and turned in *every* direction! And long enough too! Delightful contemplation.

As "to what purpose have we practised for years," if *malgre*, occasionally some tortuous, erratic root persists in not leaving its socket with crown, or fellow—why, I think that by the same rule, with equal consistency one might anathematize physicians, because they do not cure every case of cholera, or yellow fever. "Wherein is he benefitted by having his tooth fractured at the neck?" &c. Without pretending to recommend this practice, I would say that, in a case of acute ondontalgia, where such unavoidable accident followed the attempt at extraction, and brought away the nervous pulp, as probably most dentists have demonstrated in their practice, the *benefit* is about equivalent to the entire extraction of the tooth; as I should suppose the Recorder need not be informed.

ERRATA.—Several errors of print I have corrected:

1st Art.—Vol. V, No. 1, page 32, line 10,—for "statute" read statue.

2d, Art.—Vol. VII, No. 1, page 41, line 8,—insert *enough* after substance. Page 42, line 4, of case V.—for "buceal" read buccal. Page 44, last line,—for "os" read of. Page 45, line 2,—insert *it* after this.

3d Art.—Vol. VII, No. 4, page 216, line 2 of Case IX,—for "at" read *aet.* Page 217, line 3, from bottom,—insert *upon* after attempt. Page 219, line 12 from bottom,—for "bleed" read bled.

